# Brain-derived neurotrophic factor modulation of Kv1.3 channel is disregulated by adaptor proteins Grb10 and nShc

**DOI:** 10.1186/1471-2202-10-8

**Published:** 2009-01-23

**Authors:** Beverly S Colley, Melissa A Cavallin, KC Biju, David R Marks, Debra A Fadool

**Affiliations:** 1 Department of Biological Science, Programs in Neuroscience and Molecular Biophysics, The Florida State University, Tallahassee, Florida, USA

## Abstract

**Background:**

Neurotrophins are important regulators of growth and regeneration, and acutely, they can modulate the activity of voltage-gated ion channels. Previously we have shown that acute brain-derived neurotrophic factor (BDNF) activation of neurotrophin receptor tyrosine kinase B (TrkB) suppresses the *Shaker *voltage-gated potassium channel (Kv1.3) via phosphorylation of multiple tyrosine residues in the N and C terminal aspects of the channel protein. It is not known how adaptor proteins, which lack catalytic activity, but interact with members of the neurotrophic signaling pathway, might scaffold with ion channels or modulate channel activity.

**Results:**

We report the co-localization of two adaptor proteins, neuronal Src homology and collagen (nShc) and growth factor receptor-binding protein 10 (Grb10), with Kv1.3 channel as demonstrated through immunocytochemical approaches in the olfactory bulb (OB) neural lamina. To further explore the specificity and functional ramification of adaptor/channel co-localization, we performed immunoprecipitation and Western analysis of channel, kinase, and adaptor transfected human embryonic kidney 293 cells (HEK 293). nShc formed a direct protein-protein interaction with Kv1.3 that was independent of BDNF-induced phosphorylation of Kv1.3, whereas Grb10 did not complex with Kv1.3 in HEK 293 cells. Both adaptors, however, co-immunoprecipitated with Kv1.3 in native OB. Grb10 was interestingly able to decrease the total expression of Kv1.3, particularly at the membrane surface, and subsequently eliminated the BDNF-induced phosphorylation of Kv1.3. To examine the possibility that the Src homology 2 (SH2) domains of Grb10 were directly binding to basally phosphorylated tyrosines in Kv1.3, we utilized point mutations to substitute multiple tyrosine residues with phenylalanine. Removal of the tyrosines 111–113 and 449 prevented Grb10 from decreasing Kv1.3 expression. In the absence of either adaptor protein, channel co-expression reciprocally down-regulated expression and tyrosine phosphorylation of TrkB kinase and related insulin receptor kinase. Finally, through patch-clamp electrophysiology, we found that the BDNF-induced current suppression of the channel was prevented by both nShc and Grb10.

**Conclusion:**

We report that adaptor protein alteration of kinase-induced Kv1.3 channel modulation is related to the degree of direct protein-protein association and that the channel itself can reciprocally modulate receptor-linked tyrosine kinase expression and activity.

## Background

**Figure 1:**
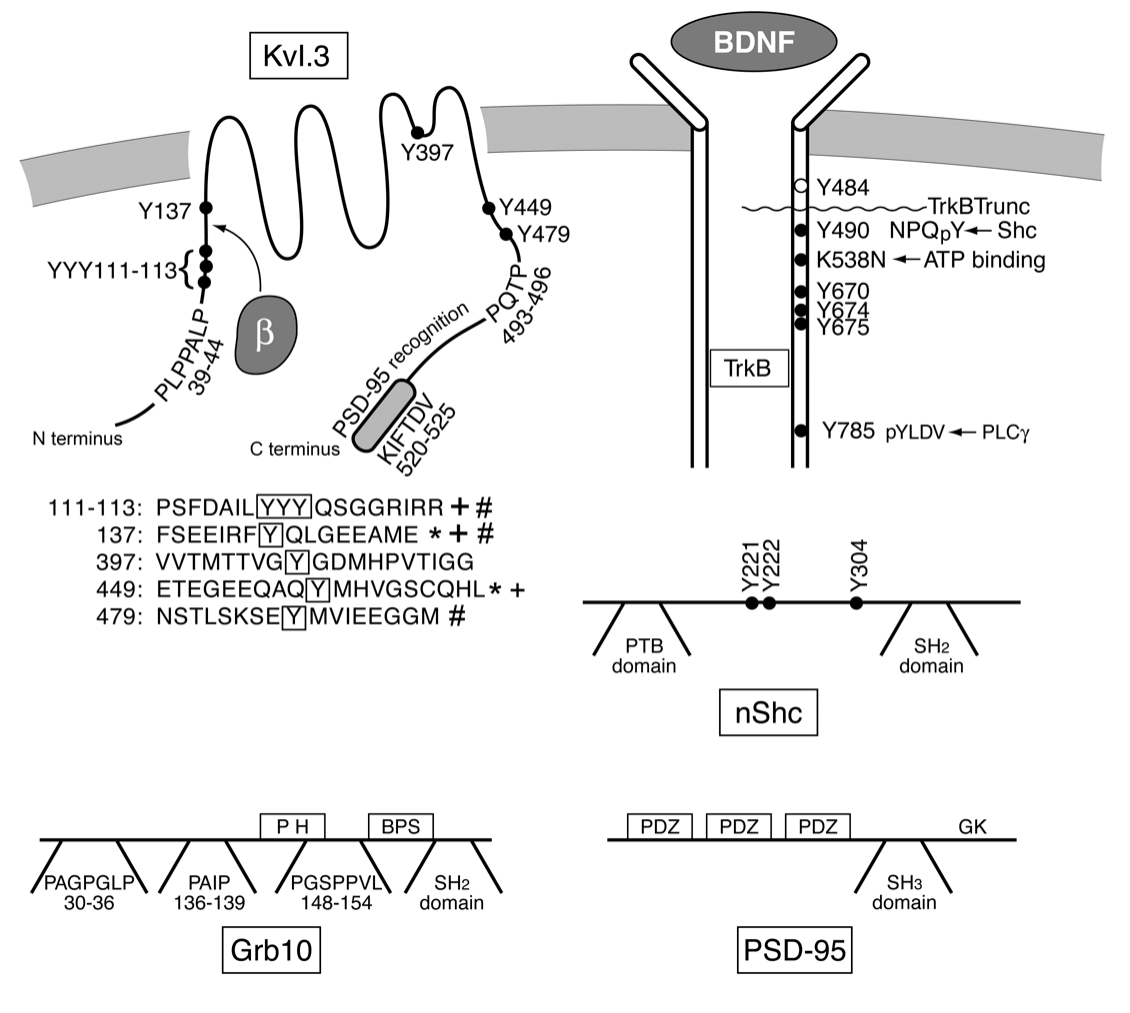
**Cartoon schematic of important regulatory structures in Kv1.3 ion channel, Neurotrophin receptor tyrosine kinase B (TrkB), and the adaptor proteins, growth factor receptor binding protein (Grb10) and neuronal and Src homology and collagen (nShc)**. Brain-derived neurotrophic factor (BDNF) binds to TrkB, which induces receptor dimerization, autophosphorylation of multiple Y residues in the β chains, and subsequent phosphorylation of the Kv1.3 downstream substrate. Site-directed mutagenesis has been used to map the molecular targets for phosphorylation of the channel via activation of insulin receptor kinase (+), Src kinase (*), or TrkB (#). ● = Tyrosine (Y) to phenylalanine (F) point mutations made to eliminate various phosphorylation recognition motifs. Proline-rich PXXP domains in channel and adaptors as noted that are known to bind to SH3- domain containing proteins. Note SH2 domains of Grb10 and nShc that recognize Y-phosphorylated residues in protein partners. Shc binding site on TrkB (Y^490^) is also noted (TrkBShc-). SH3 = Src homology 3 domain, SH2 = Src homology 2 domain, PTB = protein tyrosine binding, PH = pleckstrin homology domain, BPS = binding phosphorylated substrate, PLC = phospholipase C, β = beta subunit.

Voltage-dependent potassium (Kv) channels are regulators of neuronal excitability. The channels are responsible for maintaining the resting potential of cells, they determine the width and maximum amplitude of the action potential, and they govern the interpulse interval or timing patterns of firing in order to relay sensory information to the brain or coordinate motor output [1,2]. As recently reviewed by Kaczmarek [3], Kv channels may lead a "double life" by possessing non-conducting functions, which allow them to participate in coupled biochemical reactions or communicate directly with cytoplasmic and nuclear signaling pathways. Kv1.3, a member of the *Shaker *subfamily of Kv channels, is particularly well poised to participate in multiple cell signaling pathways given a number of molecular motifs that serve as protein-protein interaction domains in the N and C terminal aspects of the channel protein (Fig. 1). Multiple and different combinations of tyrosine residues become phosphorylated upon activation of cellular and receptor tyrosine kinase signaling cascades to elicit changes in ion channel current magnitude, inactivation kinetics, or cumulative/use-dependent inactivation [4-9]. Further levels of complexity exist, however, because the channel is not just a substrate for tyrosine phosphorylation. The phosphorylated tyrosine residue(s) now become recognition motifs for a variety of src homology 2 (SH2) domain containing proteins [10] that are down-stream signaling molecules, or adaptor proteins, without catalytic activity [11], and which functionally subserve to "modulate the modulation" [12,13]. Two proline-rich domains have been shown to serve as phosphorylation-independent binding domains for src homology 3 (SH3) containing proteins and ubiquitin ligases to change Kv1.3 channel clustering and surface expression [14]. Gene-targeted deletion of Kv1.3 demonstrates that the channel's associated scaffold of kinases and adaptor proteins becomes unbalanced in the absence of the channel gene, whereby there is a significant increase in the expression of these modulatory cell signaling proteins, including neurotrophin receptor tyrosine kinase B (TrkB), the cellular kinase Src, the adaptor proteins 14-3-3, neuronal Src homology and collagen (nShc), postsynaptic density protein-95 (PSD-95), and the growth factor receptor-bound protein 10 (Grb10) [15]. Because Kv1.3 protein has an abundance of molecular modules for protein-protein interactions (Fig. 1), it is not surprising to discover a diversity of non-conducting functions following targeted deletion of the core of the scaffold (the channel protein) [15,16].

This study focuses upon two adaptor proteins enriched in the olfactory bulb, namely Grb10 and nShc. The adaptor protein Grb10 is a member of a superfamily of adaptor proteins that also includes Grb7, Grb14, and Mig-10 [17]. The characterization of conserved molecular architecture of this family has shown that members all contain a C-terminal SH2 domain, a central segment containing a pleckstrin homology domain (PH domain), and a principle binding region for association with insulin-like growth factor receptor type 1 that also acts to stabilize insulin receptor kinase (IR) binding to the SH2 domain (Fig. 1). The N terminal domain of Grb10 is less well conserved but contains multiple PXXP domains that serve as putative binding sites for SH3 domain containing proteins [18] (Fig. 1). While Grb10 was originally discovered to be an interacting protein with the epidermal growth factor receptor (EGF-R) [19,20] and later found to be a negative regulator of the IR via ubiquitination [21-24], it may also interact with other RTKs. Grb10 has been linked with human growth retardation and type 2 diabetes when upregulated or mutated [25,26], which alters Grb10 protein-protein interactions. The adaptor protein nShc belongs to a family that includes three major genes, ShcA (Shc), ShcB (Sck), and ShcC (nShc, or neuronal Shc). All Shc isoforms possess two distinct domains that bind phosphotyrosine containing sequences, namely, the phosphotyrosine binding domain (PTB) and the SH2 domain, and a central domain (called CH1) that contains multiple tyrosine phosphorylation sites [27,28] (Fig. 1). nShc is specifically enriched in adult brain and has been found to direct survival and phenotypic maturation of neurons via association with neurotrophin receptors [29-32]. More recently, nShc has been also implicated in the modulation of hippocampal synaptic plasticity via modulation of the NMDA receptor, independent of neurotrophin receptor signaling [33].

Previous site-directed mutagenesis studies in heterologous expression systems, have demonstrated that BDNF-activation of TrkB kinase phosphorylates Kv1.3 at three tyrosine (Y) residues or clusters in the N- and C-termini of the channel, Y^111–113^, Y^137^, Y^479^, to cause suppression of current magnitude without changes in channel inactivation or deactivation kinetics [9]. Our current strategy was to co-express channel and kinase with one of two adaptor proteins that varied in their reported interactions: (1) an adaptor protein that was not known to directly associate with the neurotrophin receptor or the channel (Grb10), and (2) an adaptor protein that was known to bind to Y^490 ^(NPQpY motif) of the neurotrophin receptor (nShc). Unexpectedly, we discovered that nShc adaptor proteins reportedly characterized to interact with RTKs also used Kv1.3 channel for a binding partner directly. Grb10 did not interact with either the channel or kinase, but could indirectly modulate the channel by changing its surface expression. In native OB, however, both adaptor proteins co-IP with Kv1.3 and TrkB kinase. Our data suggest that there is a complex regulation between the channel, the kinase, and adaptor proteins such that the expression of Kv1.3 down regulates that of TrkB kinase, while the expression of Grb10 likely acts to induce ubiquitination of the channel thus decreasing channel surface expression.

## Results

### Co-localization of adaptor proteins and Kv1.3 channel in the olfactory bulb

**Figure 2:**
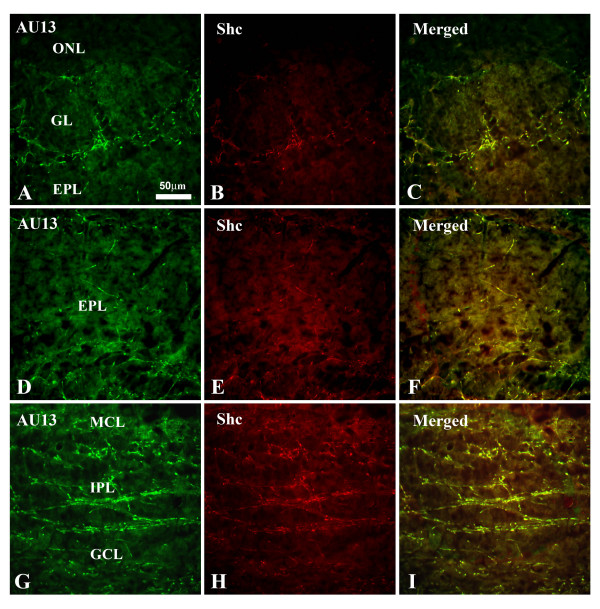
**Co-localization of nShc and Kv1.3 in the olfactory bulb of postnatal mice**. Ten micron coronal cryosections were sequentially, double immunolabeled with α AU13 (Kv1.3 antiserum, 1:500) and αShc (1:500), and visualized by incubation in species-specific fluorophore-conjugated secondary antibodies where the green channel represents Kv1.3 channel, the red channel represents Shc adaptor, and the merged image (co-localization of proteins) is yellow. Note that the channel and adaptor overlap in the peripheral structures surrounding the glomeruli and in the external plexiform layer (EPL), the mitral cell layer (MCL), the internal plexiform layer (IPL), and the granule cell layer (GCL). ONL = outer nerve layer.

**Figure 3:**
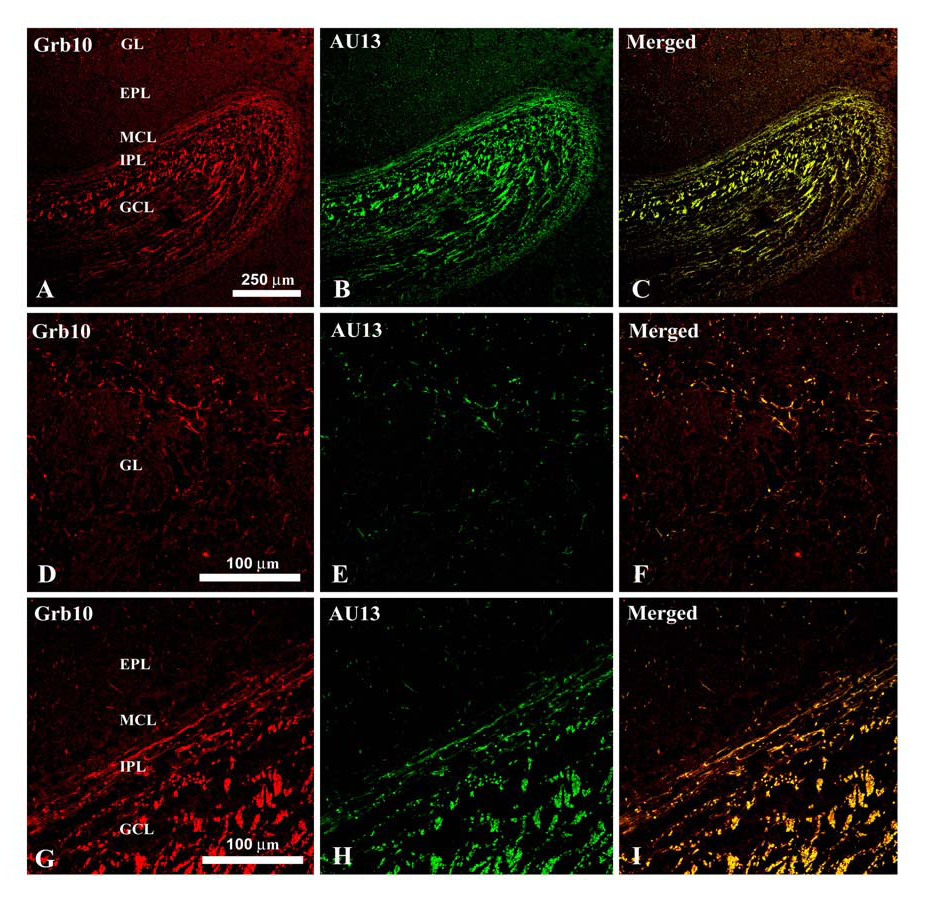
**Co-localization of Grb10 and Kv1.3 in the olfactory bulb of postnatal mice**. As in Fig. 2 with the substitution of αGrb10 (1:500). Note that the channel and adaptor overlap mainly in the IPL, GCL, and slightly in some fibers surrounding the glomeruli.

Although the goal of our study was to define the molecular targets for interaction of Kv1.3 plus TrkB kinase in the presence of adaptor proteins using a well-defined heterologous expression system (HEK 293 cells), it was important to ascertain the physiological significance of such interactions by first exploring the potential co-localization of the adaptor proteins with the channel in native brain. Kv1.3 has a limited distribution in the central nervous system, where it has been studied primarily in regions of highest expression, namely in the olfactory bulb (OB), the olfactory cortex, and the dentate gyrus of the hippocampus [34]. We have previously reported the expression of several adaptor proteins (nShc, Grb10, 14-3-3, and PSD-95) in cytoplasmic lysates of the OB by Western blot analysis [12,15] as well as the co-localization and co-IP of the channel and TrkB kinase in the OB and olfactory cortex [35]. Thus, we explored co-localization of Kv1.3 with that of nShc (Fig. 2) and Grb10 (Fig. 3) in the OB of postnatal day 20–30 mice where Kv1.3 channel properties are well characterized [9,36] and it is known that modulation of Kv1.3 alters olfactory threshold [15]. Kv1.3 and nShc demonstrated greatest overlap of expression in the peripheral structures surrounding the glomeruli (Fig. 2A–C) and weaker co-localization patterns in the external plexiform layer (EPL), the mitral cell layer (MCL), the internal plexiform layer (IPL), and the granule cell layer (GCL)(Fig. 2D–I). Kv1.3 and Grb10 demonstrated greatest overlap of expression in the IPL and GCL (Fig. 3A–C) and weaker co-localization patterns in some fibers surrounding the glomeruli (Fig. 3D–I).

### Adaptors can directly co-IP with Kv1.3 channel independent of TrkB kinase

**Figure 4:**
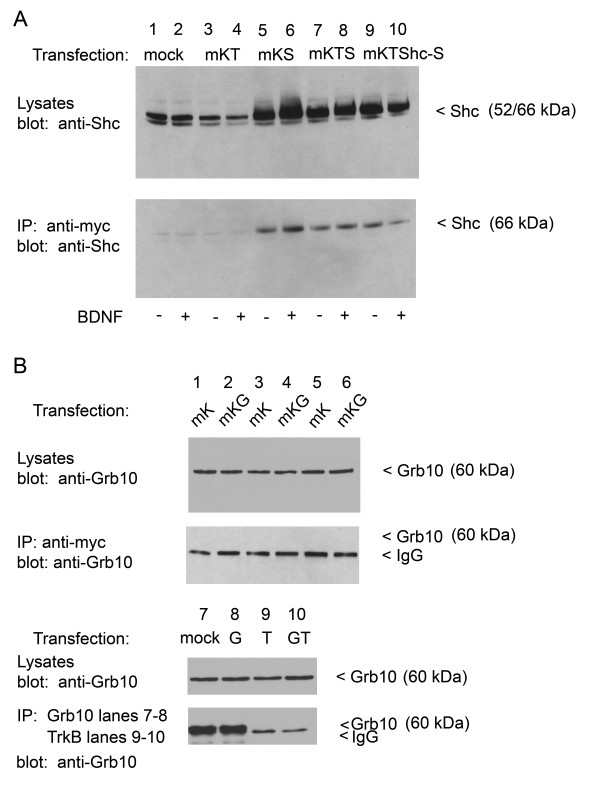
**nShc adaptor forms a protein-protein interaction with Kv1.3 channel**. (A) HEK 293 cells were transiently transfected with cDNA encoding myc-tagged Kv1.3 (mK) plus TrkB (T) or nShc (S) or triply transfected with channel, kinase, adaptor combination using either wild-type (T) or mutant TrkB kinase (TShc-) lacking the Shc binding site. Forty-eight hour (h) post-transfection, cells were stimulated with 1 ng/ml BDNF (+) or vehicle control (-) for 10 minutes at 37°C. Five to 10 μg of whole-cell lysates from each respective transfection condition were separated by SDS-PAGE, electro-transferred to nitrocellulose, and probed with αShc (anti-Shc). Upper panel shows representative expression bands for three such experiments in which endogenous (lanes 1–4) versus transfected levels (lanes 5–10) of Shc protein are compared. In the lower panel, lysates were immunoprecipitated (IP) with an antiserum against the myc epitope to pull down Kv1.3 channel protein (anti-myc), separated by SDS-PAGE and nitrocellulose was probed with αShc (anti-Shc). Note direct co-IP of Shc and Kv1.3 (lanes 5–6) is independent of BDNF stimulation. Addition (lanes 7–8) and loss of TrkB Shc binding site (lanes 9–10) does not affect the co-IP. Lower panel expression bands are representative of three such experiments. The predicted molecular mass (M_r_) for Shc is 46/52/66 kDa. (B) Similar experimental paradigm, protocol, notation, and sample size as in (A) but for Grb10 adaptor. Grb10 = G. Top paired panels show three repetitions in which myc-tagged Kv1.3 is transfected alone (mK) or including Grb10 (mKG). Lysates were probed with αGrb10 (anti-Grb10) to compare endogenous versus transfected levels of Grb10. IP of the channel (anti-myc) followed by probing with αGrb10 (anti-Grb10) fails to indicate any channel/adaptor complex. Bottom paired panels demonstrate that αGrb10 can effectively be immunoprecipitated (IP: Grb10, blot Grb10, lanes 7–8) but that it also does not co-IP with TrkB (lanes 9–10). M_r _for Grb10 is 60 kDa.

Since both adaptors demonstrated a degree of colocalization with Kv1.3 in native brain tissue, we sought more rigorous conformation for a potential protein-protein interaction using HEK293 cells in which site specific mutation of the channel could be performed to map the molecular targets for channel-adaptor scaffolding. We have previously demonstrated that BDNF-induced Kv1.3 current suppression is mediated through tyrosine phosphorylation of three sites in the N and C termini of the channel, Y^111–113^, Y^137 ^and Y^449 ^[9]. Since both nShc and Grb10 have SH2 domains that could target any or a combination of these tyrosine residues upon BDNF-induced TrkB phosphorylation, we wanted to test whether either adaptor protein could associate with the channel under BDNF-stimulated conditions. As demonstrated in Fig. 4A (lanes 5–6), immunoprecipitation of myc-tagged Kv1.3 (IP anti-c-myc), followed by Western analysis and blotting for Shc (blot anti-Shc) labels predominantly a single 52 kDa band in HEK 293 cells that were only co-transfected with mycKv1.3 + Shc (no TrkB). Therefore, the channel and the Shc adaptor protein form a protein-protein interaction complex independent of BDNF-induced phosphorylation of the channel or at least in the absence of TrkB kinase. Inclusion of TrkB kinase or Y490F TrkB (TrkBShc-) cDNA into the transfection scheme does not prevent or increase the channel/adaptor interaction (Fig. 4A, lanes 7–10), further exemplifying the lack of requirement of TrkB in the channel/adaptor complex. As shown in Fig. 4B, we were not able to co-immunoprecipitate TrkB and Grb10 (lanes 9–10) or Kv1.3 and Grb10 (lanes 1–6), indicating that unlike nShc adaptor protein [29,37], Grb10 does not (tightly) associate with the kinase. Also, note the expression of native levels of adaptor proteins, apparently endogenous to HEK 293 cells in lanes untransfected with Shc or Grb10 cDNA (Fig. 4A, lanes 1–4, Fig. 4B, lane 7).

### Adaptor/kinase/channel scaffold can be precipitated from olfactory bulb and hippocampus

**Figure 5:**
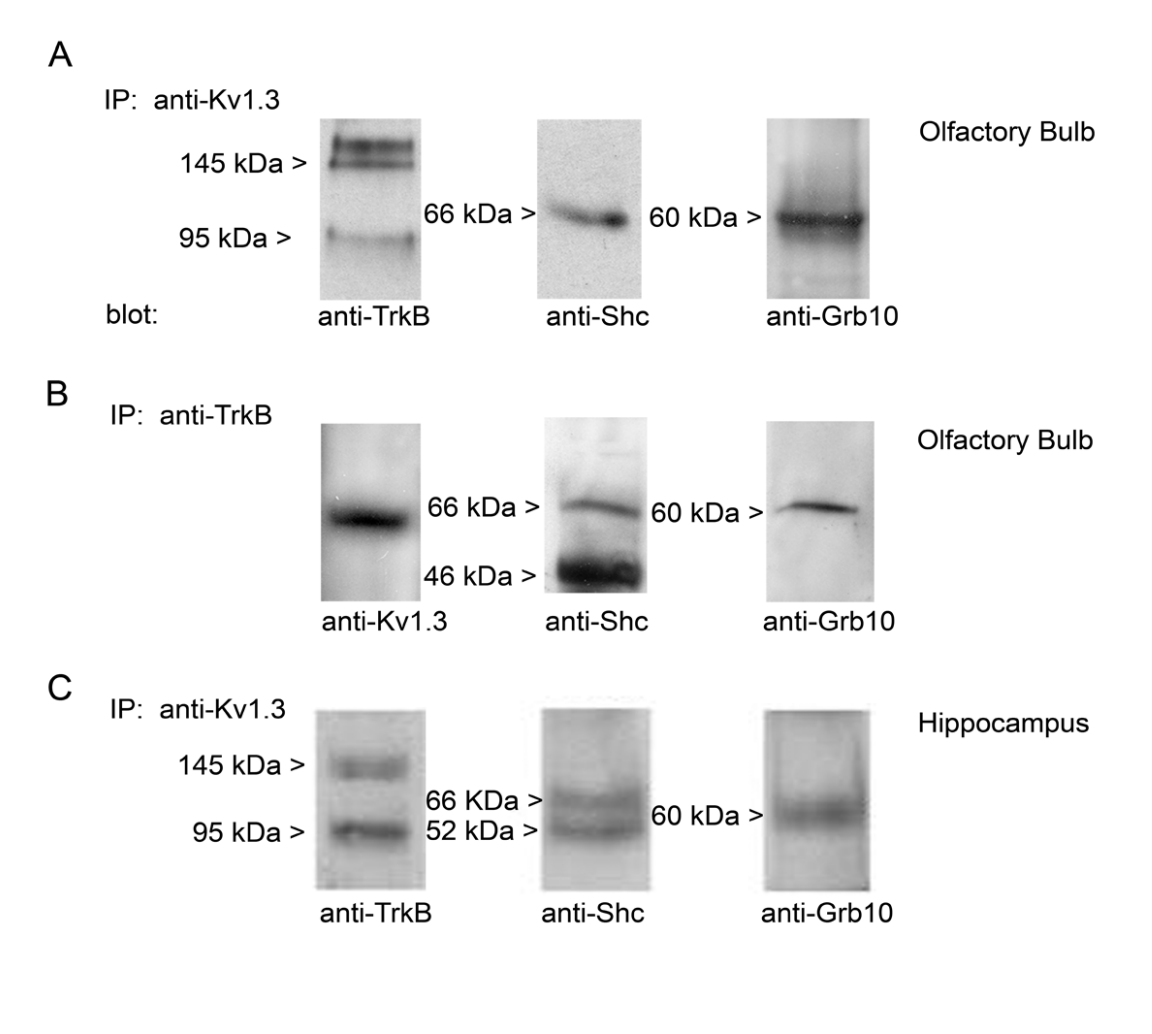
**Kv1.3 channel forms protein-protein interactions with both adaptor proteins in the olfactory bulb and hippocampus**. (A-B) Olfactory bulb or (C) hippocampus lysates were immunoprecipitated with either anti-Kv1.3 (IP: anti-Kv1.3) or anti-TrkB (IP: anti-TrkB), separated by SDS-PAGE, and Western blots were probed (blot) with channel, kinase, or adaptor antisera as noted. M_r _as specified.

Given our ability to co-immunoprecipitate nShc and Kv1.3 channel, we next explored whether the proteins interacted in native tissue by using two regions known to express Kv1.3 [34]. As demonstrated in Fig. 5, immunoprecipitation of Kv1.3, followed by Western analysis and blotting for anti-TrkB, anti-Shc, or anti-Grb10, labels protein bands of predicted molecular mass for the kinase, and adaptors respectively. As we have previously reported for the olfactory bulb [36], TrkB labeling demonstrated a greater intensity for the full-length receptor protein (140–145 kDa) over that of its truncated form (90–95 kDa) that lacks a kinase domain (Fig. 5A). The predicted molecular mass for Shc is multiple depending upon the variant (46/52/66 kDa) and in previous cytosolic fractions we have observed all three variants in olfactory bulb preparations [15]. It is interesting that in the HEK 293 cells, the molecular migration was closest to the 52 kDa variant, whereas in the olfactory bulb, the variant associated with Kv1.3 (66 kDa) was different than those associated with TrkB (46/66 kDa) (Fig. 5B). Moving to the hippocampus, the variants (52/66 kDa) forming a protein-protein interaction with Kv1.3 were also different than that in the olfactory bulb (Fig. 5C). It is also noteworthy, that while Grb10 could not be consistently co-immunoprecipitated with either the kinase nor the channel via heterologous expression (Fig. 4B), both were complexed with the adaptor protein in the olfactory bulb and the hippocampus (Fig. 5). It is possible in the HEK 293 cells that the interaction is at the limit of our detection. It may also suggest either a a missing scaffolding component in the HEK 293 cells that is essential for protein-protein interactions with the Grb10 adaptor or a different regulation of Grb10 in neurons.

### Grb10 decreases surface expression density of the channel

**Figure 6:**
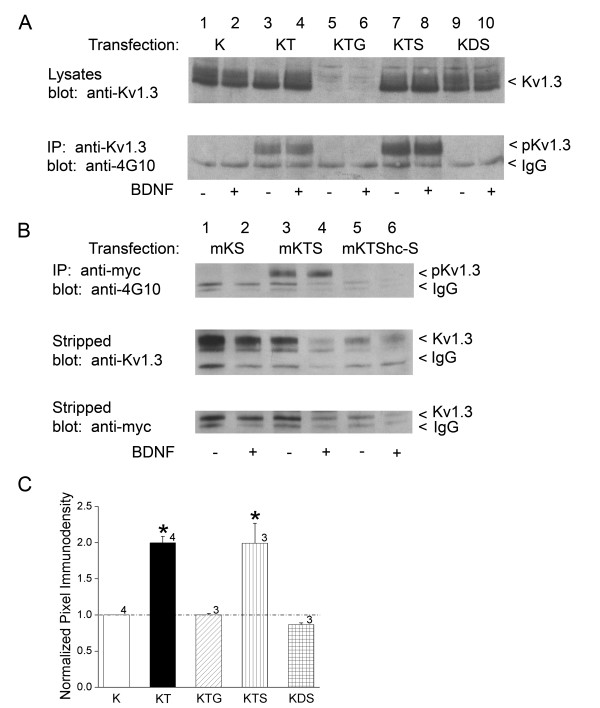
**Grb10 adaptor decreases TrkB-induced phosphorylation of Kv1.3 while nShc adaptor increases channel phosphorylation**. (A) HEK 293 cells were transiently transfected with cDNA encoding Kv1.3 (K) or Kv1.3 plus TrkB kinase (T) with either Grb10 (G) or nShc (S). A dead TrkB kinase (D) where the catalytic β chain was truncated (see Fig. 1, TrkBTrunc) was also incorporated into the transfection design (KDS). Cells were stimulated with vehicle (-) or BDNF (+), and lysates were prepared and separated by SDS-PAGE as in Fig. 4. Upper panel shows representative expression bands for three such experiments in which the nitrocellulose was probed with αAU13 to recognize Kv1.3 channel protein (anti-Kv1.3). Note increase in Kv1.3 expression with TrkB co-expression and decreased Kv1.3 expression in the Grb10 co-transfection condition. Lower panel lysates were IP with αAU13 (anti-Kv1.3) and then nitrocellulose was probed with an antiserum that recognizes tyrosine phosphorylated proteins (anti-4G10) to quantify the degree of channel phosphorylation (pKv1.3). The M_r _for Kv1.3 ranges from 55 to 72 kDa depending upon extent of phosphorylation (< Kv1.3). (B) Same experimental paradigm, protocol, notation and sample size as in (A) but substitution of myc-tagged Kv1.3 (mK) to quantify fraction of pKv1.3 at the surface membrane as opposed to total cellular channel protein. HEK 293 cells were transiently transfected with cDNA encoding myc-tagged Kv1.3 (mK) plus nShc (S) and wildtype TrkB (T) or mutantTrkB kinase (TShc-) lacking the Shc binding site. Upper panel lysates were IP with αmyc and then nitrocellulose was probed with α4G10 (anti-4G10) to quantify degree of channel phosphorylation (pKv1.3). Note loss of pKv1.3 in lanes 5–6 (upper panel) without the Shc binding site, despite expression of the channel in both whole cell fraction (middle panel) and membrane fraction (lower panel). (C) Bar graph plot of the mean ± S.E.M. normalized immunodensity values of the pKv1.3 labeled band for experiments demonstrated in A. Pixel density ratios (dashed line, 1.0 no difference) were generated for BDNF stimulated (+) conditions by normalizing the pixel density for a transfection condition (KT, KTG, KTS, or KDS) to that of control K transfection condition (K) within a single autoradiographic film to eliminate variability introduced by differential exposure times. * = significantly different compared with control K transfection condition, by Student's *t*-test, arc sine transformation for percentage data, α ≤ 0.05.

We have previously reported that Kv1.3 channel modulation can be perturbed by adaptor proteins via disruption of src- or insulin-induced Kv1.3 phosphorylation [12,13]. In the cellular tyrosine kinase signaling pathway (src kinase), adaptor proteins that do not disrupt tyrosine phosphorylation have no effect on channel function; however, those that decrease tyrosine phosphorylation can relieve kinase-induced current suppression. In the receptor tyrosine kinase signaling pathway (IR), adaptor proteins that disrupt tyrosine phosphorylation relieve both current suppression and simultaneously act to increase the rate of channel inactivation. For the neurotrophin pathway (a different receptor tyrosine kinase), we questioned how the degree of channel phosphorylation as modified by an adaptor protein might be linked to function. Thus, we immunoprecipitated Kv1.3 channel plus TrkB kinase under various transfection conditions that included either Shc or Grb10 adaptors (Fig. 6A). Interestingly, inclusion of nShc in the transfection scheme (Kv1.3 + TrkB + nShc; KTS) increased the Y phosphorylation of the channel over that observed under the co-transfection condition alone (Kv1.3 + TrkB; KT) (Fig. 6A, bottom gel, lanes 7–8 versus lanes 3–4). Moreover, elimination of the Y^490 ^TrkB site via substitution with TrkBShc- in the channel/kinase/adaptor transfection scheme (Fig. 6B; mKTShc-S, top gel, lanes 5–6) completely prevented tyrosine phosphorylation of the channel. Basal phosphorylation of Kv1.3 (- BDNF conditions) was higher than expected or reported in our previous studies [9,35], but may be attributed to the solution used as a vehicle carrier (patch solution) that may have slightly depolarized the cells (Fig. 6A, lanes 3 versus 4). None the less, all basal phosphorylation could be completely eliminated in the absence of TrkB (Fig. 6A, bottom gel, lanes 1–2) or via substitution with a dead TrkB kinase construct in which the catalytic domain was truncated (Fig. 6A, bottom gel, lanes 9–10) [37]. Most interestingly, inclusion of Grb10 in the transfection scheme (Kv1.3 + TrkB + Grb10) not only completely eliminated the Y phosphorylation of the channel over that observed in the co-transfection condition (Kv1.3 + TrkB)(Fig. 6A, bottom gel, lanes 5–6 versus lanes 3–4), blotting the lysates with anti-Kv1.3 to control for expression of the channel, revealed that Grb10 was greatly reducing the expression of Kv1.3 (Fig. 6A, top gel, lanes 5–6). The degree of BDNF-induced Kv1.3 phosphorylation in each transfection condition was quantified by densitometry, normalized to that of the Kv1.3 channel transfection alone, and statistically compared in the bar plot of Fig. 6C (Student's *t*-test, arc sine transformation for percentage data, α ≤ 0.05).

### Expression levels of Grb10, Kv1.3, and TrkB are cross-regulated

**Figure 7:**
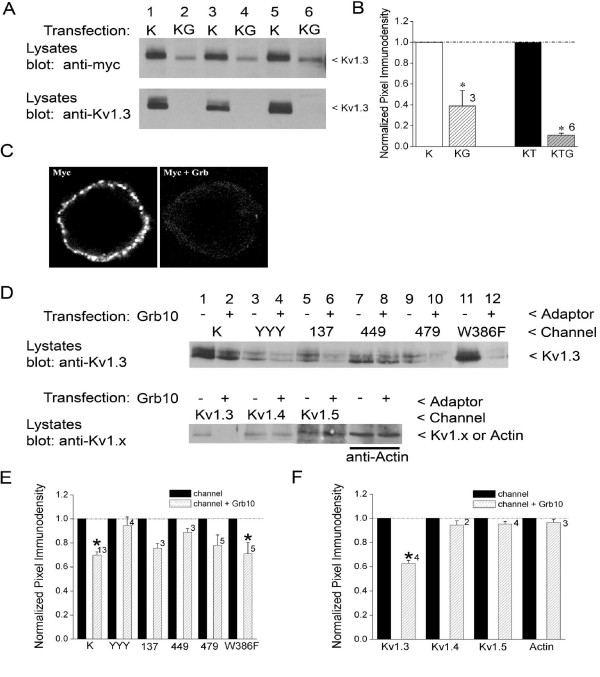
**Grb10 protein-protein interactions with Kv1.3 causes decreased channel expression that require multiple Y residues**. (A) HEK 293 cells were transiently transfected with cDNA encoding Kv1.3 (K) or Kv1.3 plus Grb10 (G) using either myc-tagged Kv1.3 (Upper panel) or wildtype Kv1.3 (Lower panel). Lysates were prepared and separated by SDS-PAGE as in Fig. 4. The M_r _for Kv1.3 ranges from 55 to 72 kDa depending upon extent of phosphorylation (< Kv1.3). (B) Bar graph plot of the mean ± S.E.M. normalized immunodensity values of the Kv1.3 labeled band for experiments demonstrated in A and also transfection schemes including TrkB kinase (KT, KTG). Pixel density ratios (dashed line) were generated by normalizing to control K transfection condition within a single autoradiographic film to eliminate variability introduced by differential exposure times. * = significantly different compared with control K or KT transfection condition, respectively, by Student's *t*-test, arc sine transformation for percentage data, α ≤ 0.05. (C) Representative HEK 293 cells (of 30) transiently transfected with myc-tagged Kv1.3 (Myc) alone or plus Grb10 (Myc + Grb10). Channel protein was detected by incubation with anti-myc under non-permeabilizing conditions and then visualized with species-appropriate fluorescent-conjugated secondary antiserum. (D) (TOP) As in (A) using wildtype (K) or various Y to F point channel mutations or a W to F point channel mutation (W386F Kv1.3); transfection scheme was minus (-) or plus (+) Grb10 adaptor. The M_r _for Kv1.3 ranges from 55 to 72 kDa depending upon extent of phosphorylation (< Kv1.3). (BOTTOM) As in (A) using Kv1.3 or two other Shaker subfamily members; transfection scheme was minus (-) or plus (+) Grb10 adaptor. NOTE: The Western analyses were performed in pairs for each channel member and therefore are not aligned to a single migration standard; Mr for Kv1.3 (55 to 72 kDa), Kv1.4 (74 kDa), Kv1.5 (64 kDa), and actin (42 kDa) varied. (E) Bar graph plot of the mean ± S.E.M. normalized immunodensity values for experiments demonstrated in (D, TOP). Pixel density ratios (dashed line) were generated as in Part (B) above, by normalizing to control transfection condition without Grb10 and comparing within a single autoradiographic film to eliminate variability introduced by differential exposure times. * = significantly different compared to minus Grb10 treatment by Student's *t*-test, Arc sine transformation for percentage data, α ≤ 0.05. (F) Bar graph plot of the mean ± S.E.M. normalized immunodensity values for experiments demonstrated in (D, BOTTOM). Analyses and statistical metric as in E.

To further explore the robustness of our finding of Grb10 downregulation of Kv1.3, we compared the surface fraction of Kv1.3 channels (mycKv1.3) and the total Kv1.3 pool (Kv1.3) in the presence and absence of Grb10. As shown in Fig. 7A, B both the surface fraction of Kv1.3 channels as well as the total pool of Kv1.3 is dramatically down regulated by Grb10 adaptor protein (quantitative densitometry, significantly-different Student's *t*-test, α ≤ 0.05). Grb10 also had the capacity to down regulate Kv1.3 protein expression in the presence of TrkB kinase (Fig. 7B). In conjunction with the ability to downregulate total channel protein, immunocytochemical experiments demonstrated a significant reduction in fluorescent signal at the membrane under Grb10 co-transfection conditions (Fig. 7C). Albeit lack of Grb10/Kv1.3 co-immunoprecipitation *in vitro*, if Grb10 was downregulating the expression of Kv1.3 channel via the SH2 recognition domains on Kv1.3, then substitution of Y to F mutations in the N- and C-terminal aspects of the channel should completely eliminate any reduction in expression. Single point mutagenesis at the selected Y residues of this study does not alter expression of the derived channel mutants as determined by either Western analysis or electrophysiology [7]. Western analysis of HEK 293 cells in which Grb10 was co-transfected with wild-type or various Y to F channel mutants revealed that residues YYY^111–113 ^and Y^449 ^represent important residues for regulation between channel and adaptor protein (Fig. 7D and 7E). Removal of these residues prevented Kv1.3 downregulation by Grb10 co-transfection. Mutation of residues Y^137 ^and Y^479 ^retained the trend for down-regulation of Kv1.3, thus these residues are likely not involved in the adaptor's regulation of channel expression. Next, we tested whether channel conductance was necessary for Grb10 down-regulation of Kv1.3 by substituting the pore-conducting mutant W386F Kv1.3 [38,39]. As shown in Fig. 7D and 7E, mutation of the pore had no effect in the adaptor's ability to down-regulate Kv1.3 channel expression. Finally, we tested whether the Grb10 down-regulation of Kv1.3 was specific to this particular *Shaker *subfamily member or could be extended to other potassium channels. Substitution in the transfection scheme of either Kv1.4 or Kv1.5, two family members that are also expressed in olfactory bulb neurons [15], elicited no change in channel expression when co-transfected with Grb10 (Fig. 7D and 7F). Furthermore, expression of general structural proteins (actin) also demonstrated no change in expression in conditions of Grb10 transfection.

**Figure 8:**
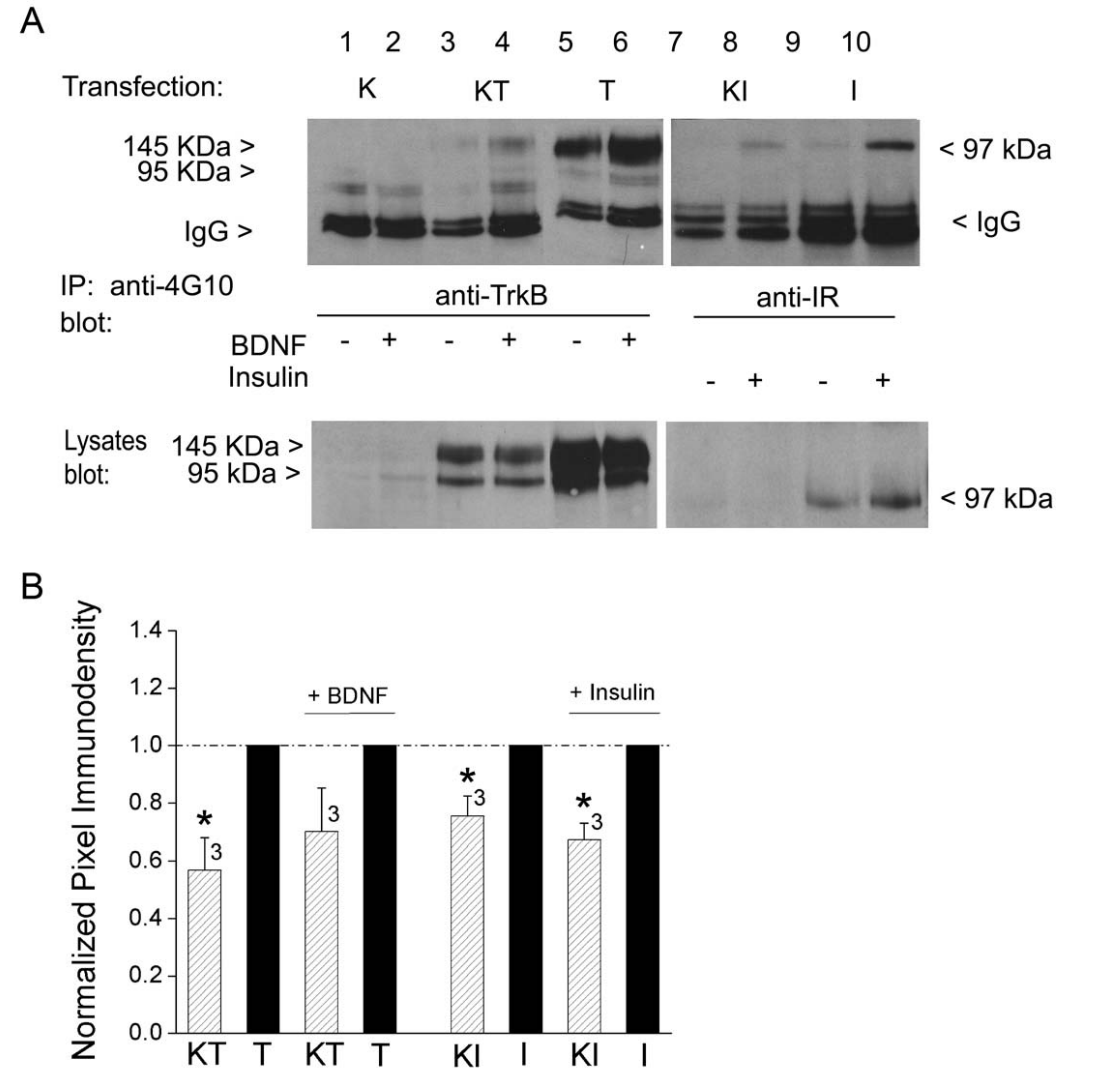
**Kv1.3 channel co-transfection suppresses receptor tyrosine kinase (RTK) expression and concomitant phosphorylation**. (A) HEK 293 cells were transiently transfected with cDNA encoding TrkB kinase (T) or Insulin receptor kinase (I) minus or plus Kv1.3 channel (KT, KI). Cells were stimulated with vehicle (-) vs. BDNF or insulin (+), and lysates were prepared and separated by SDS-PAGE as in Fig. 4. Upper panel lysates were IP with antiserum that recognizes tyrosine phosphorylated proteins (anti-4G10) and then were blotted with either αTrkB or αIR to quantify the degree of kinase phosphorylation (pIR or pTrkB). Lower panel shows representative expression bands for three such experiments in which nitrocellulose was probed with either αTrkB or αIR to recognize the respective RTK. (B) Bar graph of the mean ± S.E.M. normalized immunodensity values for either TrkB or IR kinase expression in the presence (striped bar) or absence (solid bar) of Kv1.3 co-expression under unstimulated or hormone/trophic factor stimulation (+ BDNF or + Insulin), respectively. Pixel density ratios (dashed line) were generated by normalizing kinase expression levels with the channel (KT or KI) to that of control transfection condition without Kv1.3 (T or I) and comparing within a single autoradiographic film to eliminate variability introduced by differential exposure times. * = significantly different compared to kinase alone transfection condition by Student's *t*-test, Arc sine transformation for percentage data, α ≤ 0.05.

Adaptor proteins Grb10 and nShc, respectively decreased and increased the expression of Kv1.3 channel. Of noteworthy regulation, curiously it was observed that co-transfection with Kv1.3 channel protein decreased both the expression of TrkB (Fig. 8A, bottom gel, lanes 3–6) as well as the phosphorylated state of the kinase when stimulated with BDNF (Fig. 8A, top gel, lanes 3–6). This was not only true for TrkB, but also was observed for a different, receptor tyrosine kinase (TK). Expression of Kv1.3 channel also decreased the expression of IR kinase protein and its concomitant phosphorylation by insulin (Fig. 8A, left lanes 7–10). The degree of Kv1.3 expression in the presence and absence of TrkB or IR kinase was quantified by densitometry, normalized to that of the Kv1.3 channel transfection alone, and statistically compared in the bar plot of Fig. 8B (Student's *t*-test, arc sine transformation for percentage data, α ≤ 0.05). These data pose a complex balance between channel, kinase, and adaptor whereby the level of an adaptor protein could down or upregulate expression of the channel, which could in turn, dial in the degree of phosphorylation of the kinase (that would be regulating its own neuromodulation).

### BDNF-stimulated channel modulation is blocked by Grb10 and nShc adaptor proteins

**Figure 9:**
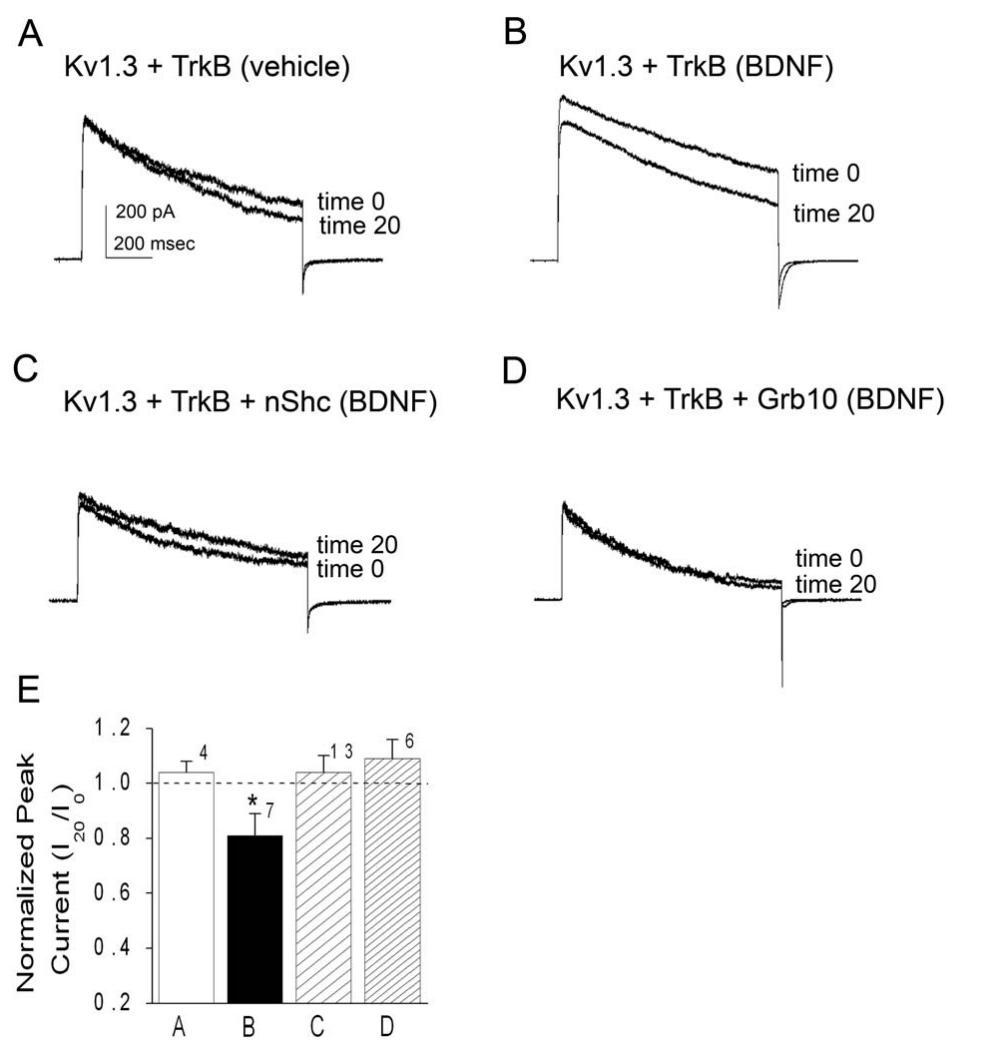
**Brain-derived neurotrophic factor (BDNF)-induced activation of TrkB causes current suppression of Kv1.3 current that is blocked by adaptor proteins nShc or Grb10**. (A-D) Human embryonic kidney 293 (HEK293) cells were transiently transfected with cDNA encoding Kv1.3 channel plus TrkB kinase with modified conditions as noted. Macroscopic currents were recorded in the cell-attached configuration by holding patches at (V_h_) = -90 mV and stepping to a single depolarizing potential of +40 mV (V_c_) for 1000 ms at an interpulse frequency of 45 s. Recording pipettes were back-filled with 1 ng/μl BDNF (BDNF) or control solution (vehicle) and tip-filled with control patch solution to monitor change in kinase activation over time. Time 0 = represents current evoked upon stable seal formation, Time 20 = post-neurotrophin treated patch 20 minutes later. (E) Histogram plot of the peak current amplitude for various transfection conditions and recordings as in (A-D). Data are normalized as a ratio of final current over initial current (I_20_/I_0_) and are expressed as mean ± standard error of the mean (S.E.M.); sample size as indicated. * = statistically significant by paired *t*-test at α ≤ 0.05. Dashed line = ratio of 1.0 or no change post-neurotrophin stimulation.

If the two adaptor proteins differentially co-immunoprecipitated with Kv1.3 channel, one might predict a functional difference in their ability to modulate the channel as determined through standard patch-clamp electrophysiology. HEK293 cells were thus transiently transfected with Kv1.3 plus TrkB kinase in the presence or absence of nShc or Grb10 adaptor protein and monitored for modulation of biophysical properties during a twenty minute acute stimulation with brain-derived neurotrophic factor (BDNF). Approximately 40 hours post-transfection, macroscopic outward currents were recorded in the cell-attached configuration. Cells were voltage-clamped at -90 mV and stepped to a depolarizing potential of +40 mV for 1 second to activate Kv1.3. Table 1 reports Kv1.3 peak current amplitude, inactivation time constant (τ_inact_), deactivation time constant (τ_deact_), voltage at half-activation (V_1/2_), and voltage dependence (κ) for a population of recorded patches under various transfection conditions pre (time 0) and post (time 20 minutes) growth factor application. Twenty minute application of BDNF had no effect on patches transfected with Kv1.3 channel alone (Table 1). This control confirms that application of ligand alone in the absence of the receptor (TrkB kinase) fails to modulate Kv1.3. Inclusion of Kv1.3 plus TrkB significantly increased peak current amplitude without altering kinetic properties (Table 1). Peak current amplitude was 287 +/- 49 pA (n = 4) in the Kv1.3-only control transfection condition and 551 +/- 198 pA (n = 4) in the Kv1.3 plus TrkB kinase condition, confirming a statistically significant 90% increase in Kv1.3 current in the presence of the kinase (Student's *t*-test, α ≤ 0.05). We have previously reported that TrkB kinase co-expression in the absence of BDNF stimulation increases the channel half-life at the plasma membrane [35] so the increase in Kv1.3 current was as expected. Secondarily, these records demonstrate typical stability of the patch over the recording interval for the co-transfection condition (time 0 = 551 +/- 198 pA versus time 20 = 557 +/- 183 pA; Fig. 9A, Table 1). Inclusion of 1 ng/μl BDNF in the patch electrode (see methods for back-fill protocol) evoked a significant suppression of peak current amplitude of Kv1.3 to 85% of its original current (Fig. 9B; Table 1, paired *t*-test, α ≤ 0.05) that was relieved by co-transfection of either adaptor protein (Fig. 9C–D; Table 1). In addition, we noted that initial peak current amplitude (time 0) of Kv1.3 in the co-transfection condition (Kv1.3 plus TrkB kinase; 555 +/- 173 pA (n = 7)) was significantly less in the presence of nShc (343 +/- 71 pA (n = 13)) but was unchanged in the presence of Grb10 (488 +/- 114 (n = 7)) (Table 1). Regardless of the pre-stimulation peak current, inclusion of either adaptor protein, prevented BDNF-induced Kv1.3 current suppression. Fig. 9E is a histogram representation of the normalized (time 20/time 0) BDNF-induced Kv1.3 current suppression (mean ± standard error of the mean (s.e.m.)) that is relieved in the presence of Shc or Grb10 (* = significantly-different paired *t*-test, α ≤ 0.05, N = 4–13). For Grb10, the relief in BDNF-induced current suppression appeared to be coincident with a slight shift in Kv1.3 voltage dependence. There was a 5 to 10 mV left shift in the voltage at half-maximum activation (V_1/2_) in the presence of TrkB plus Grb10 (but not nShc) as calculated from a Boltzman fit of the tail currents when cells were held at -90 mV and stepped to +20 mV in increments of 5 mV using a pulse duration of 50 ms (Table 1). Because this was not a robust effect and was somewhat variable even in the absence of an adaptor protein, this biophysical property was not further studied.

**Table 1:** The effect of adaptor proteins on TrkB kinase-induced Kv1.3 current properties.

Transfection Condition	Modulator	Peak current (pA)	τ_Inact_ (msec)	τ_Deact_ (msec)	V_1/2_ (mV)	κ
Kv1.3	BDNF					
Pre		287 ± 49 (4)	794 ± 252	31.5 ± 5.4	-38.2 ± 5.8	4.2 ± 0.7
Post		303 ± 68	961 ± 263	38.3 ± 13.2	-41.5 ± 6.8	4.9 ± 1.1
Kv1.3 + Grb10	BDNF					
Pre		506 ± 271 (4)	774 ± 110	31.3 ± 4.5	-39.4 ± 6.1	5.4 ± 0.4
Post		498 ± 246	*610 ± 127	34.2 ± 4.7	-41.5 ± 6.8	4.4 ± 2.6
Kv1.3 + nShc	BDNF					
Pre		543 ± 176 (7)	770 ± 45	32.6 ± 4.3	-45.0 ± 2.5	4.3 ± 0.6
Post		586 ± 107	817 ± 54	31.4 ± 4.0	-41.4 ± 3.3	3.9 ± 0.9
Kv1.3 + TrkB	Vehicle					
Pre		551 ± 198 (4)	788 ± 186	37.2 ± 3.3	-40.4 ± 5.0	5.1 ± 0.5
Post		557 ± 183	742 ± 130	32.3 ± 3.3	-38.6 ± 5.8	5.4 ± 0.7
Kv1.3 + TrkB	BDNF					
Pre		555 ± 173 (7)	900 ± 243	36.8 ± 2.9	-36.2 ± 4.6	5.8 ± 1.1
Post		*442 ± 173	762 ± 151	38.8 ± 6.7	-40.3 ± 6.7	5.3 ± 1.4
Kv1.3 + TrkB + Grb10	BDNF					
Pre		488 ± 114 (7)	862 ± 110	29.3 ± 3.5	-42.6 ± 3.7	6.7 ± 0.6
Post		545 ± 133	*553 ± 46	35.4 ± 5.5	*-54.1 ± 2.8	6.3 ± 1.4
Kv1.3 + TrkB + nShc	BDNF					
Pre		343 ± 71 (13)	618 ± 67	37.4 ± 3.2	-40.9 ± 1.9	4.4 ± 0.7
Post		381 ± 92	549 ± 68	36.4 ± 2.0	-43.5 ± 2.9	4.4 ± 1.1

HEK 293 cells were transfected with cDNAs encoding Kv1.3 ± TrkB ki nase ± adaptor protein. Several voltage-stimulating paradigms were completed (see "Methods") to collect the tabled biophysical properties by cell-attached patch-clamp recording. Mean values (± s.e.m.) for peak current amplitude (pA), inactivation time constant (τ_Inact_), deactivation time constant (τ_Deact_), voltage at half activation (V_1/2_), and slope (κ) of the voltage dependence were compared across various transfection conditions at the start of the patch recording (Pre) versus twenty minutes following 1 ng/μg BDNF (Post) using a paired *t*-test, α ≤ 0.05. * = Significantly different.

### nShc signaling at Y^490 ^TrkB is target for blocked modulation of Kv1.3 channel

**Figure 10:**
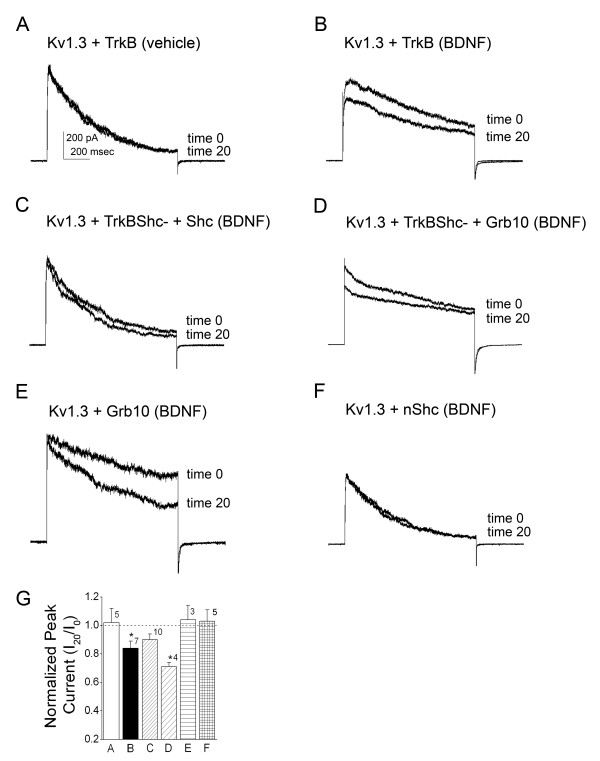
**Mutation of Y^490 ^TrkB Shc binding site differentially affects block of TrkB-induced current suppression of Kv1.3 current by adaptor proteins nShc and Grb10**. (A-F) Same experimental paradigm, notation, and analysis protocol as Fig. 9 with the substitution of mutant TrkB kinase (TrkBShc-) in which the recognition site (Y^490^) for nShc binding was altered by point mutation to F. Note that TrkB-induced current suppression observed in (B) is still blocked by co-transfection of nShc in (C) but not with Grb10 (D). Note that Grb10 co-transfection (E) but not nShc co-transfection (F) with Kv1.3 alone induced an increased rate of inactivation. (G) Histogram plot of the peak current amplitude for various transfection conditions and recordings as in (A-F). Data are normalized as a ratio of final current over initial current (I_20_/I_0_) and are expressed as mean ± S.E.M.; sample size as indicated. * = statistically significant by paired *t*-test at α ≤ 0.05. Dashed line = ratio of 1.0 or no change post-neurotrophin stimulation.

To our knowledge there no reported direct interactions between TrkB and Grb10, so we focused our study upon disruption of known targets of Shc binding to TrkB kinase. The SH2 domain (see Fig. 1) of nShc is known to bind phosphorylated Y^490 ^TrkB at the NPQpY recognition motif [40,41]. If the molecular target for nShc perturbation of BDNF-induced Kv1.3 current suppression was soley Y^490 ^on TrkB kinase, then destruction of this binding site (called TrkBShc-) should regain BDNF modulation of Kv1.3 (prevent relief of current suppression by adaptor protein). What we found by substituting the TrkB kinase mutant construct, however, was an intermediate effect. Co-transfection of Kv1.3 + TrkBShc- plus nShc cDNA exhibited BDNF-induced current suppression of Kv1.3 (533 +/- 109 pA (time 0) versus 473 +/- 115 pA (time 20) that did not reach statistical significance (not significantly different paired *t*-test, α ≤ 0.05; n = 10, 89% suppression) compared with that observed with Kv1.3 + TrkB co-expression (356 +/- 71 pA (time 0) versus 298 +/- 64 pA (time 20); significantly different paired *t*-test, α ≤ 0.05; n= 5, 84% suppression) (Table 2, Fig. 10B, C). If, however, we substituted Grb10 in the transfection scheme instead of nShc (Kv1.3 + TrkBshc- + Grb10), we observed retention of BDNF-induced current suppression that was not relieved by the adaptor protein (533 +/- 122 pA (time 0) versus 379 +/- 84 pA (time 20); significantly different paired *t*-test, α ≤ 0.05; n = 4, 71% suppression) (Table 2, Fig. 10D). Although Grb10 has never been reported to directly interact with TrkB kinase signaling cascades, and we also do not find that this adaptor co-immunoprecipitates with the kinase *in vitro *(Fig. 4B), residue Y^490 ^of TrkB kinase is an important target for nShc, and its mutation appears to somehow uncouple Grb10 modulation of the channel. With the removal of Y^490 ^of TrkB kinase, Grb10 can no longer relieve BDNF-induced current suppression of the channel (Fig. 10D and 10G). Interestingly, although neither adaptor protein induces a change in peak current amplitude of Kv1.3 alone (Fig. 10E and 10F), Grb10 also evoked a faster rate of channel inactivation (762 +/- 151 msec (minus Grb10) versus 553 +/- 46 msec (plus Grb10) as demonstrated in Fig. 10E and statistically compared in Table 1.

**Table 2:** The effect of adaptor protein signaling at Y^490 ^TrkB for Kv1.3 current properties.

Transfection Condition	Peak Current (pA)	τ_Inact_ (msec)	τ_Deact_ (msec)
Kv1.3 + TrkB			
Pre	356 ± 71 (5)	697 ± 130	32.2 ± 2.1
Post	*298 ± 64	780 ± 59	24.2 ± 2.9
Kv1.3 + TrkBShc-			
Pre	372 ± 144 (3)	693 ± 45	31.8 ± 3.3
Post	386 ± 140	843 ± 114	26.5 ± 3.7
Kv1.3 + TrkBShc- + nShc			
Pre	533 ± 109 (10)	1070 ± 163	31.2 ± 4.3
Post	473 ± 115	1040 ± 196	35.3 ± 3.8
Kv1.3 + TrkBShc- + Grb10			
Pre	533 ± 122 (4)	894 ± 173	29.0 ± 1.6
Post	*379 ± 84	660 ± 229	32.6 ± 7.8

HEK 293 cels were transfected with cDNAs encoding Kv1.3 +TrkB kinase or TrkBShc- (Y^490 ^mutation) ± adaptor protein. Several voltage-stimulating paradigms were completed (see "Methods") to collect the tabled biophysical properties by cell-attached patch-clamp recording. BDNF stimulation, notations, and statistical analysis as in Table 1.

## Discussion

Kv1.3 is a substrate for multiple tyrosine kinase signaling cascades including IR, TrkB, and src kinase [5,7-9,35,36]. Each exerts the phosphorylation of multiple tyrosine residues, and different combinations of residues, to produce a unified response, of Kv1.3 current suppression. Inclusion of adaptor proteins causes perturbation of the channel current suppression [12,13], and in the case of neurotrophin signaling, we find that disruption of the channel modulation is not directly correlated to the extent of channel phosphorylation. The scenario is more complex. This may be attributed to the fact that the interaction between kinase and channel is no longer a binary relationship, with the added adaptor having the capacity to bind to both channel and kinase to elicit different responses. Not only can an adaptor protein alter how the channel will respond to neurotrophin-induced current suppression, the adaptors alter expression levels of the channel and the channel itself can downregulate TrkB expression and its phosphorylated state. Such a dynamic equilibrium between Kv1.3/TrkB/adaptors may allow a full spectrum of modulation of channel properties dependent upon the compliment of proteins co-expressed in a neuron at a given physiological state.

Biochemically, the degree of channel phosphorylation by BDNF-induced TrkB activation is no longer directly related to the degree of functional current suppression when adaptors are added to the mix. This is very different than what is reported for the cellular tyrosine kinase, Src. Here, phosphorylation was directly linked to the degree of current suppression and one could predict the modulation of the selected adaptor protein based upon how it influenced tyrosine phosphorylation of the channel [12]. This is in stark contrast to RTKs. The addition of nShc to Kv1.3 plus TrkB transfected conditions induced an increase in both channel expression as well as BDNF-induced channel phosphorylation whereas the addition of Grb10 resulted in the opposite. Despite the fact that the adaptors evoke an opposite response biochemically, the functional ramification is identical, the prevention of BDNF-induced current suppression. In fact, nShc/Grb have structurally similar SH2 domains whereas PSD-95 lacks such phosphorylation-dependent interactive domains [13], and yet each has the capacity to functionally perturb the BDNF-induced current suppression. This might suggest that direct interaction of the adaptor and the channel may play a predominant role in preventing kinase modulation and that changed phosphorylation is a secondary effect. In the case of the two SH2 domain containing adaptor proteins, nShc and Grb10, binding of Y^490 ^TrkB by nShc is an additional key regulatory site impacting modulation of the channel. Certainly there are endogenous levels of both nShc and Grb10 expressed in HEK 293 cells, thus our Kv1.3 + TrkB conditions are not completed devoid of adaptor protein, however, when nShc is co-transfected with these conditions, binding of Y^490 ^TrkB induces a block of BDNF-induced channel suppression.

Kv1.3 channel expression is changed in the presence of TrkB kinase without BDNF stimulation and now we can also see that Grb10 adaptor significantly modifies surface expression of Kv1.3. With TrkB kinase co-expression, there is a correlate increase in channel surface expression, increase in channel protein expression, and increase in channel macroscopic current [35]. Interestingly, with Grb10 co-expression (also not BDNF dependent) there is oppositely a decrease in channel surface expression, a decrease in protein expression of the channel, but no change in channel macroscopic current, rather an increased rate of channel inactivation. The adaptor/channel do not form a complex in vitro that we can detect, nevertheless, co-expression apparently changes surface distribution and channel density but not current magnitude or conduction. This would imply that there must be MANY Kv1.3 channels that are not contributing to current. Such a pool of silent or "sleeping channels" has been reported for specific Kv subfamily members (Kv6 and Kv9 family members) [42,43] and uncovered for other potassium channels during FRAC (functional recovery after chemobleaching) [44]. Our data [35] and that reported by Sun et al. [44] support that molecular/protein density of a channel may not always exhibit linearity with functional density.

Based upon the crystal structure of Grb10 SH2 domain determined at 1.65 Å resolution, it is suggested that binding of Grb10 is dimeric, using turn containing phosphotyrosine sequences [45]. It is unknown (but doubtful) if the Kv1.3 channel contains turn containing phosphotyrosine motifs unless the tetrameric structure of the subunits permitted such an alignment, which would be contrary to positioning of the α transmembrane helices 1 and 6 for Kv1.2, a *Shaker *family member for which crystal structure has been reported [46]. On the basis of structural analysis and previous biochemical studies for the adaptor, Grb10 and Grb14 have a partially impaired ability to bind phosphotyrosine-containing ligands due to a non-glycyl residue at the end of the BD loop and lack of a P + 3 binding pocket [47]. These combined data are consistent with our findings that we cannot detect Grb10 and Kv1.3 co-immunoprecipition in vitro, albeit the fact that channel/adaptor functional interactions appear to modulate channel expression and can be found as a complex in unstimulated native tissues. The fact that mutation of several Y residues on the N and C terminal aspects of the channel clearly perturbs the negative modulatory affect of Grb10 on channel expression, indicates a specific modulatory role that does not require tight association. Moreover, the modulation does not require channel activity, or at least functional ion conductance. While all Y to F channel mutations blocked Grb10 down regulation of Kv1.3 expression, YYY111-113FFF Kv1.3 and Y449F Kv1.3 appear to be the most robust at blocking down regulation. Both of these sites (along with Y^137^) were previously reported as targets for BDNF-induced Kv1.3 current suppression and phosphorylation [9] but only Y^449 ^has a large hydrophobic M downstream of the phosphotyrosine, reported as a preferred Grb motif [48].

It has previously been reported that the E3 ubiquitin protein ligase called Nedd4-2 regulates voltage-gated ion channels, including Na_v_, Kir, and Kv family members [49] via targeting phosphotyrosine motifs located in the C-terminal aspect of the channel. It has also been demonstrated that the SH2 domains of Grb10 form a constitutive complex with Nedd4-1 so as to mediate ligand-dependent ubiquitination of IGF-IR [17]. Since co-expression of Nedd4-2 with Kv1.3 in *Xenopus *oocytes induces a strong suppression of Kv1.3 current [14] we conjecture that Grb10 might bind to endogenous Nedd4-2 in our system to cause ubiquitination of Kv1.3 and/or TrkB kinase. This might explain a potential mechanism for decreased Kv1.3 surface expression in the presence of Grb10 and correlate decreased channel tyrosine phosphorylation.

We have shown that TrkB and Grb10 act as modulators of channel expression, while others have demonstrated that the Grb10 adaptor acts as a negative modulator of IR kinase activity (reviewed by Holt and Siddle [47]). Ramos et al. [50] elucidated the mechanism by which the SH2 and binding phosphorylated substrate (BPS) domains of Grb10 might induce proteasomal degradation of the IR and subsequent inhibition of its downstream signaling pathways. The question arises as to whether the adaptor or kinases themselves are regulated and if so, how? Our data demonstrate that there is a reciprocal regulation of RTK phosphorylation activity and expression in the presence of Kv1.3. Holmes et al. [4] similarly reported that treatments that induce membrane hyperpolarization (K channel activity) markedly attenuate tyrosine phosphorylation catalyzed by cellular tyrosine kinases. Unlike their report, centralizing on reciprocal downregulation of Kv1.3 and cellular TKs, our data demonstrate that both TrkB and IR kinase protein levels are reduced when co-expressed with Kv1.3, which then in turn, decreases the total amount of kinase phosphorylation. At the same time, we also observe that both nShc and Grb10 adaptor protein expression is reduced when co-expressed with Kv1.3 (data not shown), and that the expression of both adaptors is increased in Kv1.3-null mice [15], offering another tier of regulation of the channel/kinase/adaptor interaction involving receptor-linked as opposed to cellular tyrosine kinases. Another suggested method in which Grb10 action might be terminated is via phosphorylation; in its inactive state it is thought to form tetramers [51,52]. Although we did not test whether Grb10 was phosphorylated in channel/kinase/adaptor transfected conditions under BDNF-stimulated conditions (Fig. 6), this certainly did not functionally affect downregulation of channel expression.

Grb10 appears to be promiscuous in interacting with multiple receptor and non-receptor TKs and other signaling proteins *in vitro *[47], therefore, gene-targeted deletion of the adaptor could afford physiological relevance to many of these interactions. Grb10-null mice have disproportionate overgrowth of the embryo and placenta, imbalanced glucose homeostasis, and are approximately thirty percent larger at birth [24,25,53]. This is an interesting phenotype given the fact that Kv1.3-null mice, oppositely, are resistant to diet-induced obesity, are smaller and physiologically thinner than age-matched wild-type animals, and have increased mobility and metabolism [15,54,55] – while having an increased expression of Grb10 protein that reinforces its function as a growth inhibitor [47].

In heterologous expression systems and *in vivo*, Shc adaptor protein can bind directly to the channel (where it does not alter function or expression). Shc can also bind to TrkB at Y^490 ^to increase phosphorylation of Kv1.3 and relieve BDNF-induced current suppression. To what extent is this mimicked in native olfactory bulb neurons (OBNs)? We know that exogenous application of BDNF to native OBNs [9,36], where Shc and TrkB are both present [12,15], causes an increase in Kv1.3 tyrosine phosphorylation and current suppression that is significantly greater than what we observe in heterologous expression systems [35] (typically 60% (mice) to 40% (rats) of mitral cell outward current is suppressed by acute BDNF stimulation). We also know that RTKs explicitly target Kv1.3, because in mice with Kv1.3 gene-targeted deletion, neither insulin nor BDNF has any affect on mitral cell current. Thus, there is a divergence in gain strength between HEK 293 cells and OB neurons for neurotrophic factor signaling that might lie at a missing adaptor protein not mimicked in the HEK 293 cell system, a lesser abundance of inhibitory adaptor proteins, or perhaps another regulatory molecule as yet not accounted for.

## Conclusion

In native OB, adaptor proteins may change with developmental profile, regeneration, or disease [26,28,32,56,57] – hence expression patterns of co-localization may be non-static, which would result in state-dependent channel properties. For example, it is well documented that neurotrophins are altered with injury [58-60] and that insulin is modified with metabolic state and meals [61], while both are modified with growth or development [62-64]. Therefore, the conductance through a Kv1.3 channel, which underlies the shape and frequency of the action potential output of the OB, would inherently be modified by dynamic change in physiological state. This in turn might predict that odor perception is very state dependent as dictated by its microenvironment of protein partners and adaptors that would influence a major voltage-gated ion channel in this system.

## Methods

### Solutions and reagents

Human embryonic kidney cell (HEK 293) patch solution contained (in mM): 30 KCl, 120 NaCl,10 4-(2-hydrosyethyl)-1-peperazineethanesulfonic acid (HEPES), and 2 CaCl_2 _(pH 7.4). HEK 293 cell recording bath solution contained (in mM): 150 KCl, 10 HEPES, 1 EGTA, and 0.5 MgCl_2 _(pH 7.4). Cell lysis buffer contained (in mM): 25 tris (hydroxymethyl) aminomethane (pH 7.5), 150 NaCl, 150 NaF, 0.5 EDTA, and 1.0% Triton X-100 (pH 8.0). Protease and phosphatase inhibitor (PPI) solution was added to the lysis buffer just prior to use for a final concentration as follows: 1 μg/ml pepstatin A, 1 μg/ml leupeptin, 2 μg/ml aprotinin, 10 μg/ml phenylmethylsulfonyl fluoride, and 10 mM Na_3_VO_4_. Nonidet-NP40 protease and phosphataseinhibitor (NP40 PPI) solution was prepared in the same PPI solution as above but contained (in mM): 20 Tris base (pH 7.5), 150 NaCl, 1% nonidet-NP40 and 10% glycerol. Homogenization buffer contained (in mM): 320 sucrose, 10 Tris, 50 KCl, 1 ETDA (pH 7.8). Wash buffer contained (in mM): 25 Tris base (pH 7.5), 150 NaCl, 150 NaF, 0.5 EDTA and 0.1% Triton X-100. Tris stripping buffer (TSB) contained (in mM): 10 Tris, 10 β-mercaptoethanol, with 1% SDS (pH 8.8). Sodium citrate stripping buffer (SCSB) contained (in mM): 100 Na_3_C_6_H_5_O_7 _2H_2_0, 10 β-mercaptoethanol, with 1% SDS; pH 3.0. Phosphate-buffered saline (PBS) contained (in mM): 136.9 NaCl, 2.7 KCl, 10.1 Na_2_HPO_4_, and 1.8 KH_2_PO_4 _(pH 7.4). Human recombinant brain-derived neurotrophic factor (rhBDNF) was purchased from Promega (Madison, WI). All salts and other reagents were purchased from Sigma Chemical Co. (St. Louis, MO) or Fisher Scientific (Atlanta, GA).

### cDNA constructs and antisera

All channel, kinase, and adaptor protein coding regions were downstream from a cytomegalovirus (CMV) promoter or a lac promoter. TrkB cDNA was a generous gift from Dr. P. Barker, McGill University, in a CMX vector as used previously [9]. A dead TrkB kinase (TrkBTrunc) and a targeted mutation at the Shc binding site of TrkB (Y490 TrkB or TrkBShc-) were created by Dr. David Kaplan, University of Toronto, as described previously [37] and were generous gifts to our study. Kv1.3 channel was subcloned into the multiple cloning region of pcDNA3 (Invitrogen, Carlsbad, CA) at the unique restriction *HindIII *site of the multiple cloning region. The ten amino acid c-myc epitope (EQKLISEEDL) was inserted into the extracellular S1/S2 loop between residues 226–227 of Kv1.3 channel via two consecutive polymerase chain reactions (PCRs) using the Expand Long Template PCR System (Roche, Indianapolis, IN). Both the untagged channel and epitope-tagged version of the channel were used in this study as required for surface expression and localization experiments. We have previously demonstrated that the tag does not alter channel biophysical properties or protein interactions [35]. Site-directed mutagenesis to generate Y to phenylalanine (F) at positions 111–113, 137, 449, and 479 was done as described previously [5,7]. The cDNA for a non-conducting Kv1.3 mutant with a point mutation exchanging a tryptophan (W) to F at position 386 was a generous gift from Dr. Todd Holmes (University of California Irvine, Irvine, CA) [38]. Neuronal Shc (nShc) cDNA was a generous gift from Dr. T. Nakamura (Sumitomo Electric Industries, Yokohama, Japan) and was expressed in the vector pCMV1 [29]. Grb10 cDNA was a gift from Dr. R. Roth (Stanford University) and was expressed in pBlueScript SK (-) vector (Stratagene, La Jolla, CA). Kv1.4 and Kv1.5 cDNA were both expressed in pcDNA3 and were a generous gift from Todd Holmes (University of California Irvine, Irvine, CA) [65].

AU13, a rabbit polyclonal antiserum, was generated against a 46 amino acid sequence (478MVIEEGGMNHSAFPQTPFKTGNSTATCTTNNNPNDCVNIKKIFTDV523) representing the unique coding region of Kv1.3 on the C- terminus. Genemed Synthesis (San Antonio, TX) purified the peptide and Cocalico Biologicals (Reamstown, PA) the antisera as previously characterized [36]. This antibody was used for immunoprecipitation (1:1000) and Western blot detection (1:800) of Kv1.3. Kv1.4 and Kv1.5 polyclonal antisera were used for Western blot detection (1:1000) as previously characterized and were generous gifts from Drs. J.O. Dolly and T.C. Holmes, respectively [15]. Tyrosine phosphorylated proteins were visualized by the anti-phosphotyrosine antibody 4G10 (Millipore/Upstate Biochemical; St. Louis, MO) and used at 1:1000 for Western blots. Tyrosine phosphorylated proteins were immunoprecipitated with 4G10 antibody (3 μg per 600–1200 μg of whole lysate protein). Monoclonal antiserum directed against amino acids 156–322 of human TrkB was purchased from BD Transduction Laboratories (San Jose, CA) and used at 1:1000 for Western blots. Grb10 (K-20) rabbit polyclonal antibody was purchased from Santa Cruz Biotechnology, Inc. (Santa Cruz, CA) and used at 1:800 for Western blots and at 1:500 for immunocytochemistry. Shc rabbit polyclonal antibody (cat # 610082) from BD Transduction Laboratories (San Jose, CA) was used for immunohistochemistry (1:500) and Western blots (1:750). Monoclonal actin antibody (A 2066) was purchased from Sigma and used at 1:1000 for Western blots as a secondary confirmation for equivalent protein loading. Anti-c-myc mouse monoclonal (clone E910; antigenetic peptide EQKLISEEDL against from the human myc protein) was purchased from Roche and used at 1:800 for Western blots. Donkey anti-rabbit FITC-conjugate (1:100) and goat anti-rabbit Texas Red-conjugate (1:200) were from Southern Biotech (Birmingham, AL).

### Immunocytochemistry

Procedures for surface labeling of mycKv1.3 in the presence and absence of Grb10 as expressed in HEK293 cells was as previously described [35]. Briefly, cells were labeled under non-permeabilizing conditions to image the surface distribution of the channel. Visualization and microscopic analysis was performed on a Zeiss Axioplan 2 Microscope attached to a Zeiss LSM510 confocal system (Carl Zeiss, Thornwood, NY) in conjunction with Metamorph software (Molecular Devices/Universal Imaging, Dowingtown, PA). Samples were excited at 488 nm (for GFP) and 595 nM (for Texas Red) through a 63× oil-immersion objective (NA = 1.40) and fluorescence between 500–545 and 565–615 nm was detected from 0.8 μm optical sections. Images were taken from the cells at their maximum cross section. After removing background pixels by thresholding, integrated pixel intensity was calculated both from the total cross section of the cell and from an area that encompassed the cross section of the cell excluding the outermost region.

Double-color immunofluorescence procedures for co-localization of Kv1.3 channel and either Grb10 or nShc adaptor protein in the olfactory bulb were as described in Biju et al. [16]. Briefly, C57 BL/6 mice (postnatal day 20) were anaesthetized with pentabarbitol, trancardially perfused, and post-fixed with 4% paraformaldehyde. Following sucrose cryoprotection, olfactory bulbs were coronally cut at 10 μm thickness on a Leica CM1850 microtome-cryostat (Leica, Meyer Instruments, Houston, TX). Sections were mounted on 2% gelatin coated slides and stored at -20°C until immunoprocessing. Sequential labeling was performed [16,35] using AU13 as the first primary antibody (visualized with FITC-conjugated, species-specific secondary antibody), followed by anti-Shc or anti-Grb10 as the second primary antibody, which was visualized by a Texas Red-conjugated, species-specific secondary antibody. Processed slides were viewed at 40× magnification using an Axiovert S-100 Microscope (Carl Zeiss) equipped with epifluorescence. Images were captured using an Axiocam digital camera and Axiovision associated software (v3.0.6) at a maximum pixel resolution of 1300 × 1300.

### Maintenance and transfection of HEK 293 cells

HEK 293 cells were maintained in Minimum Essential Medium (MEM), 2% penicillin/streptomycin, and 10% FBS (Invitrogen/Gibco/BRL). Before transfection, cells were grown to 100% confluency (7 days), dissociated with trypsin-EDTA (Sigma) and mechanical trituration, diluted in MEM to a concentration of 600 cells/ml, and replated on Corning dishes (Catalog # 25000, Fisher Scientific). The Corning dishes have a growing surface of approximately 8 cm^2 ^(electrophysiology) or 21 cm^2 ^(biochemistry) to favorably allow cells to divide logarithmically at the point of transfection. cDNA was introduced into HEK 293 cells with a lipofectamine reagent (Invitrogen/Gibco/BRL) 3–5 days after passage as previously described [7,8]. Briefly, cells were transfected for 4–5 hours with 0.5–0.75 μg of each cDNA construct per 35 mm dish for electrophysiology or 2–3.5 : μg of each cDNA construct per 60 mm dish for biochemistry. Plasmid DNA with no coding insert (control vector) served as the control to equalize total μg of cDNA added to each dish. Cells were either harvested for biochemical analysis or used for electrophysiological recordings approximately 30–40 hours after transfection. Efficiency of co-transfection (greater than 95%) with our transfection method has been tested using double, sequential labeling and confocal microscopic visualization, as reported previously [8].

### Immunoprecipitation and Electrophoretic Separation

Transfected cells were rinsed in PBS and then stimulated with 10 ng/μl BDNF or vehicle control solution for ten minutes at room temperature. The selection of vehicle control (patch pipette solution), temperature, and incubation time was designed to parallel conditions for biochemistry with those used for electrophysiological recordings. Cells were then harvested by lysis in ice-cold PPI solution (see Solutions and Reagents). The lysates were clarified by centrifugation at 14,000 × g for 10 minutes at 4°C and incubated for 1 hour (h) with 0.2–0.3 mg/ml protein A-sepharose (GE Healthcare Bio-Sciences Corp/Amersham-Pharmacia, Piscataway, NJ), followed by re-centrifugation to remove the protein A-sepharose. To test the phosphorylation state of Kv1.3 in the presence of BDNF-activated TrkB kinase plus or minus an adaptor protein, tyrosine-phosphorylated proteins were immunoprecipitated from the clarified lysate by overnight incubation at 4°C with 3–4 μg/ml 4G10 antibody (Millipore/Upstate Biochemical). Protein-protein interactions were also determined by a similar co-immunoprecipitation strategy by substituting 4G10 antibody for antisera directed against Kv1.3, c-myc, Shc, Grb10, or TrkB kinase. In experiments where tyrosine-phosphorylated proteins were immunoprecipitated from native olfactory bulb or hippocampus, tissues were harvested from postnatal day 20 (P20) C57BL/6 mice that had been euthanized via CO_2 _inhalation followed by decapitation according to AVMA- and NIH-approved methods. Brain regions were quickly removed from the cranium and homogenized 50 strokes by Kontes tissue grinder (size 20) in homogenization buffer on ice [8]. The lysate clarification, immunoprecipitation, and SDS-PAGE analysis were as described for HEK 293 cells above. In experiments in which surface labeled Kv1.3 was probed using c-myc antiserum, whole mycKv1.3 transfected HEK 293 cells were first incubated with c-myc antiserum (0.4 μg/μl) for 30 minutes at 37°C prior to BDNF stimulation as above. Cells with surface-labeled mycKv1.3 were then rinsed in PBS, lysed in ice-cold PPI solution. Both immunoprecipitated proteins and surface-labeled mycKv1.3 protein were harvested by a 2-h incubation with protein A-sepharose and centrifugation as above. The immunoprecipitates (IPs) were washed 3 times with ice-cold wash buffer (0.1% Triton). Lysates and washed IPs were diluted in sodium dodecyl sulfate (SDS) gel loading buffer containing 1 mM Na_3_VO_4_. Protein concentration was determined by Bradford protein assay (Biorad Laboratories, Hercules, PA). Standard curves were generated using the small percentage of Triton (final concentration of detergent = 0.001%) that did not significantly interfer with protein concentration determination in this assay. Proteins were separated on 10% acrylamide SDS gels and electrophoretically transferred to nitrocellulose for Western blot analysis. Nitrocellulose membranes were blocked with 5% nonfat milk and incubated overnight at 4°C in primary antibody against Kv1.3 or other sought protein partner. Membranes were then exposed to species-specific peroxidase-conjugated secondary (GE Healthcare Bio-Sciences Corp/Amersham-Pharmacia or Sigma) for 90 minutes at room temperature. Enhanced chemiluminescence (ECL; GE Healthcare BioSciences Corp/Amersham-Pharmacia) exposure of Fuji RX film (Fisher Scientific) was used to visualize labeled protein.

In experiments comparing Kv1.3 expression in the presence and absence of Grb10 adaptor protein, labeled Kv1.3 bands from cell lysates were quantified by densitometry using a Hewlett-Packard Photosmart Scanner in conjunction with Quantiscan software (Biosoft, Cambridge, UK). Pixel densities for each band were normalized to Kv1.3 within the same cell passage, transfection, and autoradiograph. Mean pixel densities were then calculated across sets of normalized data contained in single autoradiographs. This type of quantification was designed to reduce inherent variability in cell culture, transient transfection efficiencies, and ECL exposure times. Statistical significance was set at the 95% confidence level for immunodensitometry data that were analyzed by one-way analysis of variance (ANOVA) with Student Newman Keuls (*snk*) follow-up test. In experiments comparing Kv1.3 mutant expression in the presence of Grb10 adaptor protein, labeled Kv1.3 bands were quantified as above and normalized to the channel mutant alone condition within the same cell passage, transfection, and autoradiograph. Mean pixel densities were calculated as above, and statistical significance was set at the 95% confidence level for immunodensitometry data that were analyzed by Student's *t*-test with an arcsine transformation for percentage data.

### Electrophysiology

Patch electrodes were fabricated from Jencons glass (Jencons Limited, Bridgeville, PA), fire-polished to approximately 1 μm, and coated near the tip with beeswax to reduce the electrode capacitance. Pipette resistances were between 9 and 14 MΩ. Hoffman modulation contrast optics was used to visualize cells at 40× magnification (Axiovert 135, Carl Zeiss). Macroscopic currents in cell-attached membrane patches were recorded using an Axopatch-200B amplifier (MDS Analytical Technologies/Axon Instruments, Sunnyvale, CA), filtered at 2 kHz, digitized at 2–5 kHz, and stored for later analysis. All voltage signals were generated and data were acquired with the use of an Axon Digidata 1200 board with pClamp software (Axon Instruments). Data were analyzed using software from Microcal Origin (Northampton, MA) and Quattro Pro (Borland International, Scotts Valley, CA).

Outward macroscopic currents were recorded in the cell-attached rather than whole-cell configuration; Kv1.3 channel expression is so robust in the HEK 293 expression system that it is not routinely possible to record whole-cell currents without saturating the amplifier [4-6]. Patches were held routinely at a holding potential (V_h_) of -90 mV and stepped in 20 mV depolarizing potentials (V_c_) using a pulse duration of 1000 milliseconds. Pulses were generally delivered at intervals of 60 seconds or longer to prevent cumulative inactivation of the Kv1.3 channel [66]. Kv1.3 peak current amplitude, channel inactivation (τ_inact_) and deactivation (τ_deact_) kinetics, voltage at one-half maximal activation (V_1/2_), and slope of voltage dependence (κ) were measured in the presence of TrkB kinase and the presence or absence of an adaptor protein (N-Shc or Grb10). Each biophysical property was measured prior and following BDNF stimulation using a blocked (within cell patch) design as described previously [9,36]. Briefly, BDNF stimulation was accomplished by tip-filling (~0.01 mm) the patch-pipette with control solution to acquire the basal measurement (time 0) and then back filling (~35 mm) with BDNF (1 ng/μl) to acquire the post-simulation measurement (20 minutes). Each biophysical property was analyzed in the form of non-normalized data by blocked factorial, two-way ANOVA with a *snk *follow-up test at the 95% confidence level to determine any statistical difference in Kv1.3 channel function in the presence of kinase or adaptor proteins, with or without neurotrophin stimulation. For graphical representations of peak current amplitudes among different transfection conditions, measurements were normalized to the transfection condition of Kv1.3 alone within a single recording session and respective transfection set. Kinetic properties of Kv1.3 have been reported to be independent of current magnitude [66], thus peak current amplitude but not kinetic data were normalized to adjust for differences in channel expression between transfections.

Fitting parameters for inactivation and deactivation kinetics were as previously described [8]. Briefly, the inactivation of the macroscopic current, during a 1000 ms voltage step from -90 to +40 mV, was fit to the sum of two exponentials by minimizing the sums of squares using a bi- exponential function-(y = y_0 _+ A_1_*e*^-(x-x0)/τ1 ^+ A_2_*e*^-(x-x0)/τ2^). The two inactivation time constants (τ_1 _and τ_2_) were combined by multiplying each by its weight (A) and summing. The summed inactivation rate was calculated with the equation Tau Inactivation (τ_inact_) = [A_1 _* τ_1_) + (A_2 _* τ_2_)]/(A_1 _+ A_2_). The deactivation of the macroscopic current (τ_Deact_) was fit similarly but to a single exponential (y = y_0_+ A*e*^-(x-x0)/^^τ^). Tail current amplitudes were plotted in a current-voltage relationship and fit to a Boltzmann sigmoidal curve (Y = [(A_1 _+ A_2_)/(1 + *e*^(x-x0)/*dx*^)] + A_2_) to calculate the slope of voltage dependence (κ) and voltage at half-activation (V_1/2_) for Kv1.3.

## Abbreviations

ANOVA: analysis of variance; anti-4G10: phosphotyrosine antiserum; AU13: antiserum directed against Kv1.3 ion channel; BDNF: brain-derived neurotrophic factor; BPS: binding phosphorylated substrate; BSA: bovine serum albumin; cDNA: copy DNA; CH1: collagen homologous region 1; CMV: cytomegalovirus promoter; C terminus: carboxyl terminus of a protein; D: dead TrkB kinase; ECL: enhanced chemiluminescence; EGF-R: epidermal growth factor receptor; EPL: external plexiform layer of the olfactory bulb; F: phenylalanine; G: Grb10; GFP: green fluorescence protein; GCL: glomerular cell layer of the olfactory bulb; GL: granule cell layer of the olfactory bulb; G-protein: GTP-binding protein; Grb10: growth factor receptor-binding protein 10; h: hour; HB: homogenization buffer; HEK 293: human embryonic kidney 293 cells; HRP: horseradish perioxidase; IP: immunoprecipitation; IPL: internal plexiform layer of the olfactory bulb; IR: insulin receptor kinase; κ: voltage dependence; K: potassium channel; kDa: kilodalton; *Kv*: Voltage-dependent potassium channel; Kv1.3: *Kv *subfamily member 1.3; Kv1.3-/-: Kv1.3 gene-targeted deletion; MCL: mitral cell layer of the olfactory bulb; MEM: minimum essential media; Mig-10: *C. elegans *gene for cell migration; mK: myc-tagged Kv1.3; Myc: myc epitope tag; NA: numerical aperture; NGF: nerve growth factor; NPQpY motif: amino acid sequence of TrkB where nShc binds; nShc: neuronal Src homology and collagen; N: terminus amino terminus of a protein; OB: olfactory bulb; PBS: phosphate buffered saline; PBST: phosphate buffered saline + tween; PCR: polymerase chain reaction; PH: pleckstrin homology domain; PLC: phospholipase C; PPI: protease and phosphatase inhibitor; PSD-95: post-synaptic density 95 kDa protein; PTB: phosphotyrosine binding domain; PXXP: proline-rich amino acid sequence bound by SH3 domains; rt: room temperature; RTK: receptor-linked tyrosine kinase; S: nShc; Sck: ShcB; SCSB: sodium citrate stripping buffer; SDS-PAGE: sodium dodecyl sulfate polyacrylamide gel electrophoresis; SH2: Src homology 2 domain; SH3: Src homology 3 domain; Shc: Src homology and collagen; *snk*: Student Newman Keuls post-hoc analysis; Src: protein encoded by the gene of Rous sarcoma virus; τ_Deact_: deactivation time constant; τ_inact_: inactivation time constant; T: TrkB; TK: tyrosine kinase; TrkB: neurotrophin receptor tyrosine kinase B; TrkBShc: Y490F TrkB kinase (lacking Shc binding site); V_1/2_: voltage at half-maximum activation; V_c_: command voltage; V_h_: holding voltage; W: tryptophan; W386F: W386F Kv1.3 (non-conducting channel mutant); Y: tyrosine; YYY: YYY111-113FFF Kv1.3; 137: Y137F Kv1.3; 14-3-3: a signaling protein that binds phosphoserine/threonine residues; 449: Y449F Kv1.3; 479: Y479F Kv1.3; 2X, 3X, etc., fold.

## Authors' contributions

BC carried out the immunocytochemistry experiments in tissue sections, participated in the electrophysiology experiments, and conducted a share of the SDS-PAGE/Western analysis. MC conducted the SDS-PAGE/Western analysis and helped to draft the manuscript. KCB prepared the histological sections and performed the confocal imaging. DM conducted the SDS-PAGE/Western analysis on native tissues. DF conceived of the study and coordinated its design, constructed the channel mutants, conducted a share of the SDS-PAGE/Western analysis, and wrote the manuscript. All authors read and approved the final manuscript.
